# Cross-cultural adaptation of the Breastfeeding Self-Efficacy Scale Short Form (BSES-SF) modified for preterm mothers in Brazil

**DOI:** 10.1590/0034-7167-2022-0497

**Published:** 2023-11-27

**Authors:** Clarice Borges Lucas Denobi, Lorena Maria Fernandes da Silva, Gabriela Ramos Ferreira Curan, Cindy-Lee Dennis, Mônica Oliveira Batista Oriá, Edilaine Giovanini Rossetto

**Affiliations:** I Universidade Estadual de Londrina. Londrina, Paraná, Brazil; II State University of Toronto. Toronto, Ontario, Canada; III Universidade Federal do Ceará. Fortaleza, Ceará, Brazil

**Keywords:** Breastfeeding, Self-efficacy, Validation Study, Premature Newborn, Methodological Research in Nursing., Aleitamento Materno, Autoeficácia, Estudo de Validação, Recém-Nascido Prematuro, Pesquisa Metodológica em Enfermagem., Lactancia Materna, Autoeficacia, Estudio de Validación, Recién Nacido Prematuro, Investigación Metodológica en Enfermería.

## Abstract

**Objectives:**

to conduct a cross-cultural adaptation of the Breastfeeding Self-Efficacy Scale-Short Form (BSES-SF) for mothers of ill and/or preterm infants among Portuguese-speaking mothers in Brazil.

**Methods:**

a methodological study was completed, including the translation of the tool, synthesis of translations, review by experts, synthesis, reassessment of experts, back-translation, pre-test, and validation of the content. The study involved 19 participants, including a translator and experts. In addition, 18 mothers from the target population were included in the pre-test.

**Results:**

the equivalences of the opinion obtained by the committee of experts were semantic (85%), idiom (89%), cultural (86%), and conceptual (94%). The content validation coefficient (CVC) on the scale was 0.93 for clarity and understanding; 0.89 for practical relevance; 0.92 for relevance; and the average overall CVC was 0.91.

**Conclusions:**

the scale was translated and adapted to the Brazilian Portuguese language, which maintained the equivalences and confirmed the content validity.

## RESUMO

**Objetivos:**

realizar adaptação transcultural da *Breastfeeding Self-Efficacy Scale-Short Form* (BSES-SF) para mães de bebês doentes e/ou prematuros para a língua portuguesa no contexto brasileiro.

**Métodos:**

foi realizado um estudo metodológico, incluindo a tradução do instrumento, síntese das traduções, revisão por especialistas, síntese, reavaliação de especialistas, retrotradução, pré-teste e validação do conteúdo. O estudo envolveu 19 participantes, incluindo os tradutores e especialistas. Além disso, 18 mães da população-alvo foram incluídas no pré-teste.

**Resultados:**

as equivalências da opinião obtida pelo comitê de especialistas foram: semântica (85%), idiomática (89%), cultural (86%) e conceitual (94%). O coeficiente de validação de conteúdo (CVC) da escala foi de 0,93 para clareza e compreensão; 0,89 para relevância prática; 0,92 para relevância; e a média geral do CVC foi de 0,91.

**Conclusões:**

a escala foi traduzida e adaptada para a língua portuguesa do Brasil, o que manteve as equivalências e confirmou a validade de conteúdo.

## RESUMEN

**Objetivos:**

realizar la adaptación transcultural de la *Breastfeeding Self-Efficacy Scale-Short Form* (BSES-SF) para madres de niños enfermos y/o prematuros al portugués en el contexto brasileño.

**Métodos:**

se realizó un estudio metodológico que incluyó la traducción del instrumento, síntesis de traducciones, revisión por especialistas, síntesis, reevaluación por especialistas, retrotraducción, preprueba y validación de contenido. El estudio involucró a 19 participantes, incluidos traductores y expertos. Además, 18 madres de la población objetivo fueron incluidas en la preprueba.

**Resultados:**

las equivalencias de opinión obtenidas por el comité de expertos fueron: semántica (85%), idiomática (89%), cultural (86%) y conceptual (94%). El Coeficiente de Validación de Contenido (CVC) de la escala fue de 0,93 para claridad y comprensión; 0,89 para relevancia práctica; 0,92 para relevancia. El promedio general del CVC fue de 0,91.

**Conclusiones:**

la escala fue traducida y adaptada al portugués brasileño, lo que mantuvo las equivalencias y confirmó la validez de contenido.

## INTRODUCTION

Breastfeeding during the first six months is crucial for all children, especially preterm infants, as they have a higher risk of death, disease, complications, and behavioral issues than full-term infants^(1)^. Exclusive feeding with human milk is recognized not only as the ideal way to feed but also as a therapeutic intervention for preterm infants^(2)^, and the benefits of breast milk are considered more pronounced in this population^(3)^. Research has shown that the prevalence and duration of breastfeeding preterm infants are lower compared to full-term babies since mothers of preterm or sick babies face additional challenges in breastfeeding due to the specificities related to prematurity^(1-4)^.

The period of hospitalization after a preterm birth generates stress and insecurity in the family^(5)^. The clinical conditions and physiological immaturity of the preterm newborn often make the process of breastfeeding difficult; this complexity can be related to both the fragility of the baby and to the experiences of mothers, who, faced with challenges, commonly feel unable to breastfeed their children^(6-7)^.

It is known that women with high self-efficacy for breastfeeding put forth greater effort and persistence to overcome difficulties related to breastfeeding, unlike women with low levels of confidence, who tend to stop breastfeeding early. Thus, breastfeeding self-efficacy has been proven to be a protective factor for exclusive breastfeeding^(8-9)^. It can be described as the women’s confidence in successfully breastfeeding and involves vicarious experiences, the women’s past experiences and performances, verbal persuasion, and their present physiological and emotional states^(10)^. This concept was developed based on the Cognitive Social Theory proposed by Bandura, which assumes that self-efficacy is the person’s belief or confidence in his ability to complete a task or solve a particular problem^(11)^. Recognizing women’s behavior facing breastfeeding, Dennis and Faux developed the Breastfeeding Self-Efficacy Scale (BSES) to determine this behavior of maternal confidence or self-efficacy in breastfeeding^(10)^.

The BSES was initially a self-report instrument with 33 items underlined by two dimensions: breastfeeding technique and intrapersonal thoughts. Later studies compared internal consistency statistics with the original BSES’s suggested item redundancy. As such, 18 items were deleted using explicit reduction criteria, resulting in the new 14-item BSES-SF^(12)^.

Fourteen years later, Canadian nurses have developed the Breastfeeding Self Efficacy Scale-Short Form (BSES-SF) for mothers of ill and/or preterm infants based on the differences in breastfeeding patterns between preterm and full-term infants and given the specific challenges that mothers of sick or preterm babies face when initiating and continuing breastfeeding^(12)^. To date, it is the only tool developed to specifically assess the self-efficacy of mothers of preterm or ill infants worldwide.

BSES has been translated into several other languages^(13-15)^. Still, its version for mothers of ill and/or preterm infants was not translated and adapted for many different contexts. It motivated a cross-cultural adaptation of the scale to Brazil, considering the relevance and potential contribution of maternal self-efficacy assessment for breastfeeding preterm infants.

It is crucial to highlight that, in the case of this construct, confidence as a subjective feeling, the value of its understanding, measurement, and intentional approach introduces a potential for improving the results of breastfeeding that have been increasingly demonstrated in the literature.

## OBJECTIVES

To conduct a cross-cultural adaptation of the Breastfeeding Self-Efficacy Scale-Short Form (BSES-SF) for mothers of and/or preterm infants among Portuguese-speaking mothers in Brazil.

## METHODS

### Ethical aspects

The instrument’s author granted authorization via email to carry out this study, which the Ethics Committee approved in research involving human beings at the State University of Londrina (UEL). The study followed ethical precepts with electronic acceptance of the ICF by professionals and mothers, as well as confidentiality and anonymity of participants.

### Study design, place, and period

**Figure 1:**
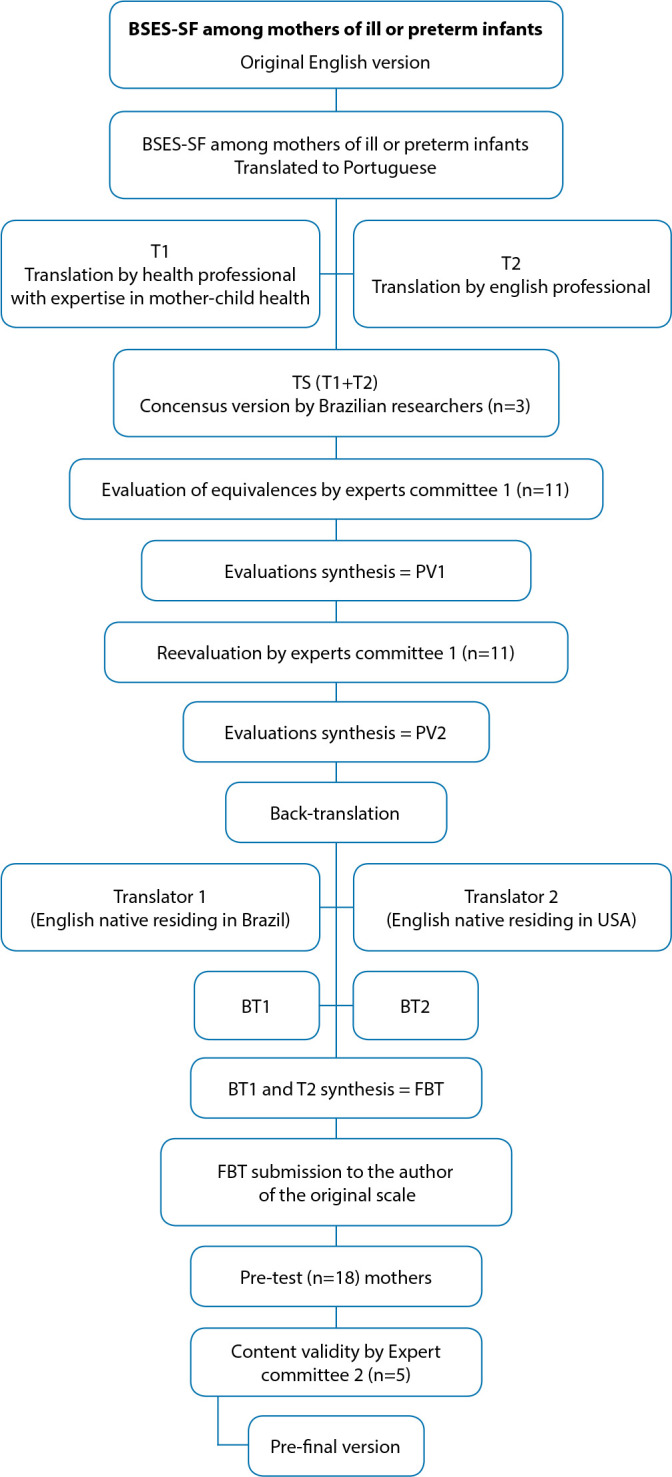
Flowchart of the translation and cross-cultural adaptation process of the Breastfeeding Self Efficacy Scale-Short Form (BSES-SF) for mothers of ill and/or preterm infants in the Brazilian Portuguese context, Londrina, Paraná, Brazil, 2022

This methodological study is based on equivalence steps^(16)^ for the cross-cultural adaptation process, carried out from May 2020 to April 2021, summarized in Figure 1.

### Population or sample; inclusion and exclusion criteria

The study participants and the inclusion criteria adopted differed according to each research stage and involved 19 participants, including a translator and health professionals. Experts were initially chosen for convenience; subsequently, a search was carried out for the lattes curriculum, privileging the region of origin and seeking to contemplate all Brazilian regions according to the inclusion criteria: health professionals with knowledge of the English language and experience in research and care in neonatology, breastfeeding, or child health. The criteria for the back translators were: English native speakers and fluency in Portuguese. One of them was an expert in the maternal-child area, while the other was a professional translator and English teacher who laid out the concepts of the study.

In addition, from January to March 2021, 18 mothers from the target population were included in the pre-test. There is no consensus in the literature regarding the minimum sample size for the pre-test; thus, we followed the approach of comparable studies that used a similar sample size. For instance, in the original development of this scale, the authors had a sample of ten mothers in the pre-test^(12)^. For translating the modified BSES-SF for mothers of preterm infants into Turkish, 12 mothers were included^(15)^, and in Chinese, 15 mothers participated in the pre-test^(9)^.

The sample of the target population was obtained by convenience, with the following inclusion criteria: mothers of preterm infants or patients with a corrected gestational age of at least 33 weeks and a maximum of 36 weeks and 6 days, who had expressed the desire to breastfeed, and who were with the baby hospitalized in the Neonatal Intermediate Care Unit (ICU-NEO) or rooming unit at the HU-UEL maternity hospital. The University Hospital of the State University of Londrina (HU-UEL), which has the certificate rooming unit of Baby-Friendly Hospital, is a public institution of regional reference in the care of pregnancy, childbirth, and high-risk newborns in five macro-regional areas in northern Paraná, with an obstetric emergency room on permanent staff.

### Study protocol

Two Portuguese native speakers initially translated the original English (BSES-SF) among mothers of ill or preterm infants to Portuguese, which originated two independent translations of the instrument (T1 and T2) from the original language (English) to the target language (Portuguese). Next, three researchers proceeded to the consensual version-synthesis of the T1 and T2 documents to overcome the discrepancies and translation difficulties. The version synthesis was sent for evaluation by a committee of 10 specialist nurses selected for convenience from different regions of Brazil (the south, southeast, and northeast).

The committee of Expert 1 received an invitation letter by email clarifying the translation and adaptation process, objective, and methodology of the study. After the acceptance, the experts also received by email the consent form, the instructions, the synthesis of the translations, the original version of the scale, and the evaluation tool provided by Google Forms^®^. The evaluation requested from the experts consisted of comparing each translated item with the original version, assigning their opinion to analyze of equivalences, and issuing comments with suggestions for improving the instrument. The experts should consider the grammatical and vocabulary evaluation (semantic equivalence), the elaboration of equivalent expressions in Portuguese for idiomatic expressions of difficult translation (idiomatic equivalence), the use of terms consistent with the cultural reality of the study population (cultural correspondence), and the translated concepts should be explored and experienced by the Brazilian population (conceptual equivalence)^(17)^.

The study analyzed the data obtained for each equivalence after the respondents filled out the form provided by Google Forms^®^. The formula used to calculate the percentage of agreement between the judges is described below^(18)^.


$$\text{ \% Agreement }=\frac{\text{ No. of participants who agreed. }}{\text{ Total no. of participants }}\times 100$$

According to the synthesis of the expert committee’s suggestions 1, the study researchers included some words and excluded those considered irrelevant, inappropriate, or ambiguous. The committee of expert 1 reviewed the changes, making up Portuguese version 1 (PV1), whose analysis obtained a concordance of 96. After final adjustments by the researchers, the Portuguese version 2 (PV2) was generated.

Two independent translators (BT1 and BT2) who did not participate in the first translation stage, both native to English and fluent in Portuguese, received the Portuguese version 2 (PV2) to prepare the back-translations. Then, the researchers synthesized the back-translations, which allowed the investigation of inconsistencies and revision of the instrument items. The resulting final back-translation version (FBT) was sent to the original author of the scale for her approval.

The pre-test with the 18 mothers was carried out from January to March 2021 to verify the clarity and understanding of the BSES-SF among mothers of ill or preterm infants to Portuguese and contribute to the semantic, idiomatic, and cultural equivalence. The researchers informed the mothers about the research aim, completed the consent form, answered the sociodemographic questionnaire, and presented the final translated scale. A subset of the sample also completed the semantic evaluation of the scale.

To assess the scale’s comprehension, the mothers answered a seven-question questionnaire on a 5-point Likert scale: 1 = Bad; 2 = Reasonable; 3 = Good; 4 = Very Good; 5 = Excellent. The questions about the evaluation of the scale addressed the following: clarity of the instructions, understanding of the items, degree of ease, understanding of the words used, clarity of the questions, and time spent to fill the scale. In addition, there was an open-ended question where participants could describe the difficulties, misunderstandings, or suggestions for making the scale more straightforward to apply.

After the pre-test, five experts formed a second committee. They had experience in preterm infant healthcare, and breastfeeding promotion, or the validation of measuring instruments. They were two speech therapists and three nurses who developed teaching, assistance, consulting, and research activities. Regarding the level of education, two had a master’s degree, and three had a Ph.D.

The researchers emailed the judges a description of the scale’s purpose, the study’s objectives, the translated scale, the consent form, and the questionnaire to evaluate the content. The purpose of this phase was to validate the content using the content validity coefficient (CVC), which encompasses the concepts of language clarity, practical relevance, and theoretical relevance, using a five-point scale (1 = totally disagree; 2 = disagree; 3 = partially agree; 4 = agree, and 5 = totally agree).

### Analysis of results and statistics

**Figure 2:**
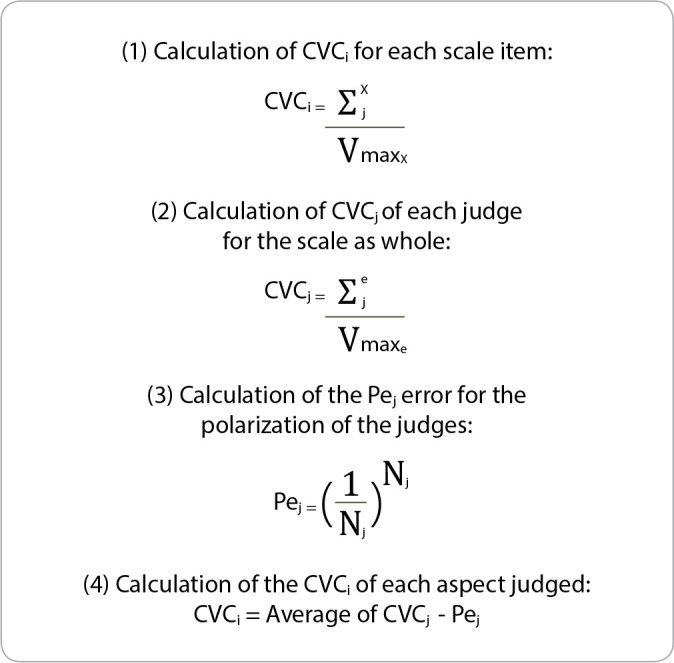
Content Validity Coefficient calculation algorithm

The content validation coefficient for each item (CVCi), the CVC of each expert judge (CVCj), and the standard error for the polarization of judges (Pej) were calculated according to the Content Validity Coefficient calculation algorithm described in figure 2 ^(19)^.

*Presentation of what was used in the present study to calculate each value of CVC (CVCi) and total CVC (CVCt) for the following studies: global construction of the translation and correspondence with the semantics of the translation item; CVC - Content Validity Coefficient(19)*

The content validation coefficient for each item (CVCi) was calculated by dividing the average of the values assigned by the expert judges (Σx j) by the maximum value of the last category of the Likert scale (Vmax) for a given item, “x.” The total CVC of the scale (CVCt) was calculated by subtracting the CVC of the expert (CVCj) from the scale by. Standard Error (Pej) of the judges’ polarization. The CVCj is the division of the total average of the scores (assigned to all items on the scale) by the maximum value of the Likert scale. Pej, in turn, is calculated by the ratio of 1 to the absolute number of judges (Nj), raised to the absolute number of judges itself^(19)^. Questions that obtained CVCi ≥ 0.80 were considered acceptable^(20)^. The study used Microsoft Excel^®^ 2016 software for data tabulation. Data are presented in tables, grouped, and complied with the experts’ suggestions when deemed relevant. After considering the recommendations of the expert judges, the study constituted a pre-final version of the tool.

## RESULTS

The variations were determined and solved by three people from the group of researchers who contributed to this study. Moreover, the averages of the adequacy of the equivalences of the opinions obtained by the committee of experts were semantics: - 85%; idiomatic: 89%; cultural: 86%; and conceptual: 94% (88% being the average of the percentage of total agreement of the equivalences).

Some items presented a concordance below 80%. However, the group of researchers decided to reformulate and improve the other items with a concordance above 80% through the following changes from qualitative suggestions: 1. replacing of words and expressions with others more applicable in the Brazilian cultural context; 2. adding words and expressions to improve understanding of sentences. In the revaluation of the scale (PV1), an average of 96% agreement was obtained among the experts, and, with suggestions for specific changes, they originated the Portuguese version 2 (VP2). The equivalence evaluation by the expert committee 1 and the consensus of the researchers were summarized below in Chart 1.

**Chart 1:** Evaluation of equivalences by experts committee 1 and Consensus of researchers, Londrina, Paraná, Brazil, 2022

Item	English term	Conflicting terms	Suggestions	Consensus of researchers
Scale title	Ill	*Doente*	No suggestions	*Doente*
Instructions	Baby Mark	*Recém-nascido* *Assinale*	*Bebê* *Marque*	*Bebê* *Marque*
Score	Confident Not very confident	*Confiante* *Não muito confiante*	*Confiante* *Pouco confiante*	*Confiante* *Muito pouco confiante*
Item 1	Pump	*Retirar*	*Extrair/Ordenhar*	*Retirar*
Item 2	Breast pumping Time consuming	*Retirada de leite* *Ser demorado*	*Extração do leite* *Ordenha do leite* *Consumir muito tempo*	*Retirar leite* *Ser demorados*
Item 3	Pumping Challenging	*Retirada de leite* *Desafiadoras*	*Extração do leite* *Ordenha do leite* *Difíceis*	*Retirada de leite* *Difíceis*
Item 4	My satisfaction	*Me sinto satisfeita*	*Que eu me sinta satisfeita*	*Que eu me sinta satisfeita*
Item 5	Keep wanting	*Continuo desejando*	*Ainda desejo* *Continuo querendo*	*Continuo querendo*
Item 6	With my	*Com minha*	*Com a minha*	*Com a minha*
Item 7	If and / or when	*Se/quando*	*Se e/ou quando* *Se eu precisar (ou quando precisar)*	*Se eu precisar (ou quando precisar)*
2ª session	To actually	-	*Efetivamente*	*Realmente*
Item 8	Determine EDF	*Determinar* *Alimentado*	*Saber* *Amamentado* *Receber leite*	*Saber* *Amamentado*
Item 10	Enough milk	*Leite suficiente*	*Leite suficiente na mamada* *Leite suficiente durante a amamentação* *Sugando adequadamente*	*Leite suficiente no meu peito*
Item 11	Able to manage to breastfeed	*Capaz de lidar com a amamentação*	*Capaz de amamentar* *Capaz de iniciar a amamentação*	*Capaz de amamentar*
Item 12	Supplement Formula	*Complemento* *Outro leite*	*Suplemento* *Leite em pó* *Fórmula infantil*	*Complemento* *Outros tipos de leite*
Item 13	Members Comfortably	*Membros* *Confortavelmente*	*Pessoas* *Tranquilamente*	*Pessoas* *Confortavelmente*
Item 14	Finish feeding Before switching to the other	*Terminar de amamentar* *Antes de mudar para o outro*	*Esvaziar o peito* *Antes de mudar para o outro peito*	*Esvaziar o peito* *Antes de mudar para o outro*
Item 15	Every feeding	*Todas as vezes*	*Todas as mamadas* *Todas as vezes*	*Em todas as mamadas*
Item 16	Demands	*Demanda*	*Demandas* *Necessidade*	*Necessidades*
Item 17	Tell	*Reconhecer*	*Dizer*	*Reconhecer*
Item 18	I will be able to switch from mostly pumping to mostly or completely breastfeeding my baby	*Eu serei capaz de trocar a retirada do leite, na maioria das vezes, pela amamentação, na maioria das vezes ou em todas as vezes, para alimentar meu bebê*	Many suggestions were put forward	*Eu serei capaz de trocar a retirada do leite das mamas pela amamentação, na maioria das mamadas ou em todas ela*s

In the pre-test, women aged between 31 and 44 years predominated (8; 44.4%), with equality between single women and those who maintain a stable relationship with their partners (9; 50%); the majority had between 7 and 11 years of education (14; 77.7%); engaged in household chores in their own homes (11; 61.7%), and served as a source of support for the family or contributed to the household budget (7; 38.8%). However, family income was low, with 8 (44.4%) families having a per capita income of at most one minimum wage (R$ 1,100.00, equivalent to US$ 217). In addition, 16.6% of women were exposed to the risks of tobacco and reported having consumed alcoholic beverages during pregnancy.

Regarding obstetric data, 12 (66.6%) women had never had an abortion, 9 (50%) had only one living child, and all had previously breastfed their children. Among women who had breastfed, 4 (22.22%) stopped at less than six months. Regarding the current pregnancy, 12 (66.6%) had 32 to 37 gestational weeks and had attended six or more prenatal consultations, and 94.4% reported the desire to breastfeed their babies.

The research applied PV2 to 18 mothers of preterm infants, who evaluated the clarity, understanding, and adequacy of the items, and 83.4% (n = 15) indicated the two best response options (very good or excellent); similarly, in the filling instructions, 94.4% (n = 17) considered them very good or excellent. 78% (n = 14) of the mothers evaluated the questions that addressed the understanding of the items and the degree of ease in answering the scale as very good or excellent. However, when asked about the comprehension of the words used, 83% (n = 15) rated them as very good or excellent, and, in item 6 (clarity and ease of answer), 94.4% (n = 17) rated them as very good or excellent. About the time spent to complete the scale, 83.3% (n = 15) judged it to be very good or excellent. In the question open to questions or suggestions, one interviewee highlighted the importance of addressing the topic.

About the content validation process, the results obtained from the individual content validation coefficient (CVCi) and the total content validation coefficient (CVCt) are described in Table 1.

**Table 1:** Calculation of the content validation coefficient of individual items (CVCi) and the total content validation coefficient of the scale (CVCt), Londrina, Paraná, Brazil, 2022

Main subject of the item	CVCi		
	Clarity	Practical relevance	Relevance
Getting enough milk by pumping	0.88	0.80	0.92
Pumping and breastfeeding are time-consuming	0.92	0.92	0.92
Dealing with breastfeeding in the same way as with other difficult tasks	0.80	0.92	0.88
Dealing with breastfeeding and feeling satisfied	0.96	0.88	0.92
Continuing wanting to breastfeed	0.96	0.88	0.96
Satisfaction with the experience	0.92	0.88	0.88
Getting help with breastfeeding	0.96	0.84	0.96
Recognizing the baby’s need to be breastfeed	0.96	0.88	0.92
Ensuring a proper latch	0.96	0.92	0.92
Knowing if the baby is getting enough milk	0.96	0.92	0.96
Breastfeeding with baby crying	0.96	0.88	0.92
Breastfeeding my baby without using formula	0.96	0.88	0.92
Breastfeeding with family members present	0.92	0.88	0.88
Feeding the baby on one breast before switching to the other breast	0.96	0.92	0.96
Breastfeeding the baby for every feeding	0.96	0.92	0.92
Meet baby’s breastfeeding needs	0.88	0.88	0.88
Recognizing when the baby has finished breastfeeding	0.96	0.92	0.92
Switching from mostly pumping to mostly or completely breastfeeding	0.92	0.92	0.92
Total	0.9148	Pej 0.00032	0.9145

*(1)CVCi - Content Validation Coefficient of Individual Items (2)Pej - Standard Error.*

## DISCUSSION

This study aimed to conduct a cross-cultural adaptation of the Breastfeeding Self-Efficacy Scale-Short-Form (BSES-SF) for mothers of ill and/or preterm infants among Portuguese-speaking mothers in Brazil. The process described reinforces the relevance of meticulous care and methodological rigor for adopting instruments created in different contexts and cultures, which should contemplate the differences in the lifestyle of the population target^(16,18-20)^.

Throughout the translation process, the study sought the equivalences between the original version and the translated version concerning the essence of the content (semantic equivalence), the concept of the evaluated phenomenon (conceptual equivalence), colloquialisms and linguistic expressions (idiomatic equivalence), and cultural experiences (conceptual equivalence), which were achieved through the evaluations of the experts and not only the literal translation of the words^(17)^. To that end, two different committees evaluated the converging and diverging points of the translations to avoid linguistic, psychological, cultural, and understanding biases^(21)^.

In evaluating the equivalences, the semantics obtained the lowest percentage of agreement among the experts, and the conceptual reached the best average. During this process, the study valued qualitative suggestions over quantitative ones, unlike the recommendation to change only items with less than 80% agreement^(22-23)^. In addition to assessing the adequacy of items considered unclear or incomprehensible, the study followed most of the experts’ suggestions, seeking a better understanding and equivalence.

The term newborn is conceptually designated to infants up to 28 days after birth. However, considering that breastfeeding is recommended exclusively until the 6th month of life and may extend up to 2 years of age or more, the study chose to replace the term “*recém-nascido*” (newborn) with “*bebê*” (baby), considering a Brazilian conceptual adaptation.

When adapting the Portuguese term *“retirar”* to “pump,” the judges suggested the Portuguese terms “*bombear”* or “*ordenhar”,* but it was considered that the word *“ordenhar”,* in the dictionary, means to extract the milk of some animals^(24)^. This expression may not be well accepted by some women who feel culturally treated as milk producer objects, such as milking machines, deprived of values, and unable to make choices. Even though man is from the class of mammalian animals, the translation should not be carried out in the literal sense semantically^(17)^. Therefore, the word *“retirar”* was chosen, and the word “mama” was included to improve the meaning and understanding of the sentence. The scientific use of the term *“extração de leite”* has been common^(25)^; however, colloquially, it might not be the most easily understood idiomatic expression. At the time of the pre-test with the target population, the participants understood and accepted this decision.

Item 18 regarding pumping for breastfeeding was questioned in all types of equivalence and obtained the lowest rate of agreement, with only 30%, and it is necessary to adjust the question almost entirely. According to the judges, the item was confusing and repetitive. Through the judge’s considerations and suggestions, the researchers opted for the final version of the item (switching from mostly pumping to mostly or completely breastfeeding), which finally reached 100% agreement.

The review and reassessment process is such a crucial translation stage and differentiates the translation methodology from others. Although studies have shown several guidelines for the cross-cultural adaptation of measuring instruments, there is still no consensus on which one is the best^(26-27)^. It should be noted, however, that the use of several reviewers allowed for a more independent contribution. This process consists of obtaining different expert opinions on a given topic through validations articulated in phases or cycles^(28-30)^, unlike consensus, where experts can influence each other.

One of the particularities of this scale is the need to apply it at different breastfeeding times and to mothers of newborns depending on different gestational ages. Considering that the study did not find any divergences among the 18 mothers interviewed, regardless of the moment and gestational age, this number of participants was considered satisfactory to evaluate semantically the version of the scale translated regarding “understanding” and “clarity” of the items, because no one reported any problems related to uncertainties or difficulty in interpretation affecting the comprehension and clarity of the scale. In addition, the target population demonstrated the acceptability of the tool, which is crucial in a cross-cultural adaptation process. The study considered the overall scale assessment by a sample of the target population positive since the majority indicated the answer options “very good” or “excellent” in all questions.

Recently, other studies have validated the BSES-SF modified for mothers of ill or preterm infants in different languages, one of which was Turkish in Izmir, Turkey, in a pilot study with 12 mothers of preterm infants to test the technical equivalence of the scale^(15)^. The other was conducted in China and was applied to 15 mothers in order to test the understanding and readability of the Chinese scale version^(9)^.

The present research used the calculation of the CVC to find numerical evidence of the judges’ agreement in the final phase of the adaptation of the tool, unlike the qualitative and subjective evaluation for translating the tool by the first committee of experts. In addition to having professional experience in the scaling topic, the second committee was multidisciplinary and included people experienced in tool validation studies. The CVCt of the scale was 0.91, and the three evaluated dimensions of the tool obtained values greater than 0.89, which attests to the validity of the tool’s contents. Similar results were found in other studies that validated the BSES-SF for Mothers of Premature Infants, with a content validity index (CVI) of 0.95 for the scale in the Chinese version, and a CVI of 93.75% the Turkish version, indicating that in both languages there was satisfactory agreement among the specialists, indicating good content validity for the scale^(9,15)^. Still, some issues, even with clarity and relevance values considered adequate, were reviewed, and modified according to the comments from the evaluating judges. Thus, after the changes, the pre-final Portuguese version of the tool was created with validated content.

### Study limitations

We attempted to follow best practices and ensure a multidisciplinary committee of bilingual experts and experts in the tool area was formed^(17-18)^. The translated scale was evaluated by professionals with the recommended qualifications from different regions and Brazilian states, considering the geographical magnanimity of Brazil and its cultural diversity, encompassing language variations among areas. However, the study did not consider all regions since it did not include judges from the North and Midwest.

The study presents some limitations concerning the translation process and cross-cultural adaptation; without them, its validity could be enhanced. The pre-test sample of the scale included only mothers of preterm infants above 33 weeks of gestational age, although the scale has the potential to be applied to mothers of sick babies who are not premature. Future work could include mothers of sick babies born at term and babies younger than 33 weeks. Pretesting of the tool was carried out in only one region of Brazil and given the significant linguistic and regional differences in the country; it is suggested that the tool be applied in the other Brazilian regions at the time of future validations. On the other hand, this limitation was minimized by the participation of judges from more than one region. The tool was tested in mothers with their hospitalized babies, so it would be interesting to verify the application of the scale in other contexts-for example, after hospital discharge.

### Contributions to Nursing Practice

The cross-cultural translation and adaptation of the modified BSES-SF for mothers of ill or preterm infants in the cultural context of Brazil may contribute to revealing the gaps in maternal knowledge and skills and, thus, provide subsidies for individualized and assertive care planning through strategies to promote maternal confidence, increasing the likelihood of breastfeeding success. The validated translation of the scale may also motivate the research using the modified BSES-SF for mothers of sick or preterm infants to evaluate the effectiveness of strategies that promote the improvement of the confidence of breastfeeding women, favoring the use of modified and innovative ways of care with this population.

## CONCLUSIONS

Considering the objectives of the research and the evidenced results, all the recommended steps were carried out for the translation and cultural adaptation of the BSES-SF. Based on expert analysis and a pretest among mothers of preterm infants, the BSES-SF maintained semantic, idiomatic, cultural, and conceptual equivalence with the original version. The content validity coefficient (CVC) of 0.91 confirmed the content validity of the tool adapted for the Brazilian Portuguese language, which corresponds to a value higher than the minimum acceptable. Although the process of translation and cultural adaptation has been finalized with validation of the content for the Brazilian context, this study recommends future studies to produce evidence of the construct validity and reliability of the tool for the entire specific Brazilian population.

## Data Availability

https://doi.org/10.48331/scielodata.GNJEJF
